# Knowledge of pregnant women about birth defects

**DOI:** 10.1186/1471-2393-13-45

**Published:** 2013-02-20

**Authors:** Ajediran I Bello, Augustine A Acquah, Jonathan NA Quartey, Anna Hughton

**Affiliations:** 1 Department of Physiotherapy, School of Allied Health Sciences, College of Health Sciences, University of Ghana, P.O.Box, KB 143, Accra, Ghana

**Keywords:** Birth defects, Knowledge, Pregnant women, Antenatal clinics

## Abstract

**Background:**

Occurrence of birth defects (BD) remains an important public health issue. Inadequate knowledge about the defects among prospective mothers could result in delayed interventions. The study determined the knowledge of BD among pregnant women in relation to their socio-demographic profile.

**Method:**

Four hundred and forty-three (443) pregnant women gave their consent to participate in this study. A researcher-administered questionnaire was used to obtain information on socio-demographic characteristics from the participants and their knowledge about BD. The questionnaire was assessed for test re-test reliability before been administered. The possible scores on the knowledge domain of the questionnaire were categorized into three levels: low knowledge (0–4), moderate knowledge (5–8) and high knowledge (9–12) levels. Data were analyzed using percentages while Spearman’s rank correlation was used to determine the relationship between the knowledge of BD among the participants and their socio-demographic profile. Alpha level was set at p < 0.05.

**Results:**

A greater proportion of the participants, 235(53.0%) were found in the age range 21 to 30 years, and 234(52.8%) attained secondary level of education. Majority of the participants, 205(46.3%) had high knowledge on the risk factors while 213(48.1%) and 224(50.6%) had moderate overall knowledge and specific knowledge about BD respectively. Most of the participants (48.1%) believed that BD were of supernatural origin. The age, level of education, number of antenatal visits and parity of the participants were not significantly correlated (p > 0.05) with their specific and overall knowledge.

**Conclusions:**

Particpants generally had moderate knowledge about BD. However, this had no bearing on their socio-demographic profile. The knowledge base about BD seems to be influenced by traditional belief of the participants. This finding should therefore serve as a guide for health care providers while planning awareness campaign about BD.

## Background

Birth defects (BD) are a significant but under-recognized cause of mortality and disability among infants and children under five years of age. The World Health Organization (WHO) defined BD as structural or functional abnormalities that are commonly seen at birth [1]. The BD is also one of the causes of spontaneous abortion and still birth and it remains a cardinal Millennium Development Goal for the year 1990–2015 targeted towards reducing mortality rate of children [2]. Children who survive and live with BD are faced with the increased risk of developing life-long physical, cognitive and social challenges on which medical intervention and other supportive services have little impact [3], hence the need to increase awareness among the expectant mothers.

About 94% of infants born with BD were reported to come from the middle and low income countries and it also recorded 95% of the death of such children from BD [4]. Although studies on the prevalence of BD is not readily available in most African countries including Ghana, a cross-sectional survey showed an incidence of 3.7% of major BD in South Western Nigeria [5]. In another report, a high percentage of women over the age of 35 years gave birth without the availability of community education, family planning services, medical genetic screening, prenatal diagnosis and other associated services in most developing countries [6].

The defects pose serious psychological stress on nursing mothers due to potential life-long disability. The problem may be compounded by the late detection and management of BD which often results from inadequate knowledge about the defects thereby undermining the health seeking behaviours of mothers after delivery [7]. For instance, due to poor knowledge of BD among the residents of an improvised area of India, children that were born with defects are abandoned and left to grow up in orphanages or in the streets [8]. Several factors have also been implicated to influence BD in pregnant women. These include nutritional deficiency, maternal illness and infections during pregnancy [9]. The previous epidemiological studies reported that genetic causes account for about 65**%** to 70**%** of total BD [10], environmental factors caused 5% to 10% [11], while obesity before or during pregnancy could lead to poor birth outcomes [12]. Given the relevance of socio-demographic profile of parents and caregivers in geographical context, its exploration among Ghanaian women with regard to BD is yet to be documented.

Adequate knowledge about BD among pregnant women could result in early detection and prompt consultation with the relevant health care professionals by nursing mothers and the care givers. The aim of this study was therefore to determine the level of knowledge about BD among Ghanaian women attending antenatal clinics at three selected health care facilities in Greater Accra Region through consideration of their socio-demographic profile.

## Methods

### Participants

Participants were recruited at the antenatal units of Korle-Bu Teaching Hospital as well as Achimota and Ridge Regional Hospitals in Greater Accra Region of Ghana. The three selected health care facilities serve as referral centers for all obstetrics and gyneacology cases in the Region. The sampled pregnant women attending the antenatal clinics were from towns and communities within the Region. They were enlisted into this study using sample of convenience. Eligibility for inclusion were; attendance in antenatal clinics at the selected Hospitals and moderate proficiency in English and Twi languages. Participants with communication challenges and those who were health care professionals were excluded.

### Instruments for data collection

A structured researcher-administered questionnaire was designed by the researchers to collect data in this study. The questionnaire was subjected to scrutiny by cross-section of experts in this field to remove all seemingly ambiguous questions. It comprised two sections: Section A sourced basic socio-demographic information of the participants such as age, number of antenatal sessions attended, level of education and parity. Section B comprised information about BD which consisted of two parts with 6 items each. The first part tested participants’ specific knowledge on the risk factors and prevention of BD whilst the second part contained items about overall knowledge of BD. Items in the second section partly contains closed ended questions such as “Yes”, “No” “I dont know”and an open ended question about their perceived reasons for the occurrence of BD. The total scores obtainable was 12 marks whereby each correct answer attracted 1 mark while incorrect answer was scored as zero (0). The scores on the knowledge domain were categorized into three levels: low (0–4), moderate (5–8) and high (9–12) levels. The test re-test reliability of the tool was assessed by administering it to 20 pregnant women prior to the commencement of the study and Chronbach’s alpha of 0.81 was obtained. The questionnaire can be used both as self-administered and researcher-administered tool (See Appendix).

### Procedure

Ethical clearance was obtained from the Ethics Review Committee of the School of Allied Health Sciences, College of Health Sciences, University of Ghana. Participants consented to take part in the study by signing the informed consent form after they had been thoroughly briefed about the purpose of the study. Five Research Assistants (RA) were involved to administer the questionnaire. They were co-opted into the study after explaining the protocol of the study to them and the questionnaire was administered to them in order to assess their responses. Each RA in turn administered the questionnaire to the Principal Researchers so as to compare their relative responses. The questionnaires were administered to all the pregnant women who registered and were attending antenatal clinics of the selected hospitals. It took RA maximum duration of about twelve minutes to administer the questionnaire to the participants.

### Data analysis

Data were analysed with the SPSS version 19.0 and Microsoft Office Excel 2007. Variables were presented using percentages. Spearman’s rank correlation coefficient was used to measure the relationship between level of knowledge of the participants and their age, educational level, parity and number of antenatal visits made. The level of significance was set at p < 0.05.

## Results

### Socio-demographic characteristics of the participants

Four hundred and forty-three (443) participants were involved in this study with majority, 235(53.0%) found in age range 21 years to 30 years. Approximately, 234(52.8%) had completed secondary school while 38(8.6%) of the participants had no formal education. Their parity ranged from 0 to 7 with 148(33.4%) being pregnant for the first time. Also, 191(43.1%) had antenatal visits not more than 10 times (Table 1).

**Table 1:** Socio-demographic characteristics of the participants

**Characteristics**	**N(n = 443)**	**%**
**Age (years)**		
<20	23	5.2
21-30	235	53.0
31-40	176	39.7
41-50	8	1.8
51-60	1	0.2
**Education level**		
Primary	100	22.6
Secondary	234	52.8
Tertiary	71	16.0
None	38	8.6
**Number of pregnancies**		
One	148	33.4
Two	124	28.0
Three	92	20.8
Four	49	11.1
Five	19	4.3
Six	6	1.4
Seven	5	1.1
**Number of antenatal visits**		
<10	191	43.1
10-19	129	29.1
20-29	69	15.6
>30	54	12.2

Participants’ specific knowledge about BD was assessed and the results are presented in Table 2. About 149(33.6%) of the participants indicated that BD is not a disease acquired by pregnant women while 380(86%) were aware that BD could be managed medically. In addition, 208(47%) indicated that physiotherapy services could have impact on the deformities that are commonly associated with BD.

**Table 2:** Participants’ specific knowledge on birth defects

**Item**	**Yes**	**No**	**I don’t know**
	**N(%)**	**N(%)**	**N(%)**
Which of the following correctly describes BD?			
It is a disease acquired by pregnant women	143(32.3)	149(33.6)	151(34.1)
It can affect baby development in the womb	275(62.1)	54(12.2)	114(25.7)
It can be transmitted by contact with affected individuals	83(18.7)	269(60.7)	91(20.6)
Most BD are preventable	330(74.5)	43(9.7)	70(15.8)
Most BD can be treated or managed medically	380(86.0)	16(3.6)	46(10.4)
Deformities from BD can be reduced through physiotherapy	208(47.0)	29(6.5)	206(46.5)

Key: Physiotherapy; BD = Birth defects.

Participants’ knowledge about risk factors and prevention of BD are presented in Table 3. The results showed that taking some unprescribed medication was the most frequently identified risk factor by 389(87.8%) respondents. Only 192(43.3%) participants identified advanced maternal age (≥40 years) as a risk factor of BD while 187(42.2%) were aware that consumption of salts fortified with iodine in their meals could reduce the incidence of BD.

**Table 3:** Participants’ knowledge about risk factors and prevention of birth defects

	**Yes N(%)**	**No N(%)**	**I don’t know N(%)**
**Risk factors**			
Will alcohol consumption during pregnancy increase your risk of giving birth to a child with BD?	324(73.1)	56(12.6)	63(14.2)
Will the use of some un-prescribed medications increase your risk of giving birth to a child with BD?	389(87.8)	32(7.2)	22(5.0)
Will smoking before and during pregnancy increase your risk of giving birth to a child with BD?	362(81.7)	26(5.9)	55(12.4)
Will advance maternal age (≥40 years) increase the risk of giving birth to a child with BD?	192(43.3)	143(32.3)	108(24.4)
**Prevention of birth defects**			
Will consumption of iodated salt during pregnancy reduce your chances of giving birth to a child with BD?	187(42.2)	97(21.9)	159(35.9)
Will regular checkups throughout the pregnancy period reduce your chances of giving birth to a child with BD?	371(83.7)	34(7.7)	38(8.6)

Key: Physiotherapy; BD = Birth defects.

Participants’ cumulative score on knowledge about BD are presented in Table 4. Most participants, 224(50.6%) had moderate scores (5–8 marks) on specific knowledge while 205(46.3%) had high scores (9–12 marks) on their knowledge with regard to the risk factors and prevention of BD. Generally, 189(42.6%) participants had high scores (9–12 marks) on the overall knowledge about BD.

**Table 4:** Participants’ cummulative scores on their level of knowledge about birth defects

**Variable**	**N**	**%**
**SPECIFIC KNOWLEDGE ON BIRTH DEFECT**		
High	127	28.7
Moderate	224	50.6
Low	92	20.7
**KNOWLEDGE ON RISK FACTORS OF BIRTH DEFECT**		
High	205	46.3
Moderate	168	37.9
Low	70	15.8
**OVERALL LEVEL OF KNOWLEDGE ON BIRTH DEFECT**		
High	189	42.6
Moderate	213	48.1
Low	41	9.3

### Relationship between participants’ level of categorical knowledge about birth defects and their socio-demographic characteristics

The relationship between participants’ categorical knowledge about BD and their socio-demographic characteristics are presented in Table 5. There were no significant relationships between the specific knowledge of the participants and their ages, (r = −0.065; p = 0.169), educational level (r = −0.112; p = 0.019), number of pregnancies (r = −0.030; p = 0.528) and the number of antenatal visits (r = 0.001; p = 0 .987). Similarly, the participants’ knowledge on the risk factors of BD was not significantly related with their socio-demographic characteristics.

**Table 5:** Spearman’s rank correlation coefficient showing relationship between participants’ categorical knowledge about birth defects and their socio-demographics

**Socio-demographic**	**Number**	**rho**	**p-value**
**Specific knowledge**			
Age	443	−0.06	0.169
Educational level	443	−0.11	0.190
Parity	443	−0.03	0.528
Number of antenatal session	443	0.01	0 .987
**Knowledge on risk factors**			
Age	443	0.01	0.787
Educational level	443	−0.01	0.256
Parity	443	−0.04	0.415
Number of antenatal session	443	0.01	0.967
**Overall knowledge**			
Age	443	−0.05	0.336
Educational level	443	−0.04	0.378
Parity	443	−0.05	0.283
Number of antenatal session	443	−0.01	0.838

In addition, there were no significant relationships between the overall knowledge of the participants and their age (r = −0.046; p = 0.336), level of education (r = −0.042; p = 0.378), number of pregnancies (r = −0.051; p = 0.283) and number of antenatal sessions attended (r = −0.010; p = 0.83).

Majority among the participants, 48.1% and 42.9% believed that BD were due to supernatural factors and medical reasons respectively. Others 31.2% and 23.7% were of the view that living an immoral lifestyle and eating some forbidden foods during pregnancy will lead to defect in a child after delivery. Another 8.1% believed that a mother is likely to have a deformed child if she gives birth to many children.

## Discussion

The focus of this study was to determine the knowledge of Ghanaian pregnant women on BD. Majority of the participants (53%) were within the age range 21–30 years, which is the usual childbearing and marriage age for most Ghanaian women. Most of the participants (68%) attained either secondary or tertiary education level, thus they were moderately proficient in English language while all had deep understanding of the local Twi language being the most widely spoken local language in Ghana, hence the ease of administration of the questionnaire. Generally, the level of education, number of antenatal visits and parity of the participants had no significant relationship with their specific knowledge, knowledge in relation to risk factors and the overall knowledge about BD.

Although 89% of pregnant women were reported to have made antenatal visits with a median visit of 4.6 in 1998 [13], the outcome of this study indicated that 191 (43.1%) of pregnant women made less than 10 antenatal visits at the time of this research. Routine interaction in medical fora such as antenatal session plays an important role in educating the public with regard to the risk of having a child with BD. Timely and regular antenatal visit is expected to significantly influence the knowledge of the pregnant women on issues relating to their eventual offspring. Based on the finding of the present study however, it could be deduced that a gap still exists in the specific education being impacted on the women during the antenatal session.

Specific knowledge of the participants on prevention, possibility of transmission and awareness of medical management for BD including physiotherapy showed that 143(32.3%) were not aware that BD is not exclusively a disease acquired by pregnant women but rather a disease that affects a fetus. Despite the above findings, 275(62.1) responded that BD can affect baby development in the womb. Generally, 168(37.9%) had moderate knowledge on the risk factors of BD. This response may probably be due to the general perception of the participants in connection with pregnancy which might have predisposed them to the awareness about preventive and precautionary measures. This background knowledge might have been transferred naturally and synchronously to the prevention of BD and may not necessarily be in connection with their knowledge about congenital deformities.

Although 380(86.0%) knew that BD was amenable to medical treatment, only 208(47.0%) of the participants knew specifically that physiotherapy could be employed to manage BD. The knowledge of the participants in this regard is inadequate considering the pivotal role of physiotherapy in the management of BD. Congenital club foot for instance could be successfully managed in 90% of patients through manipulation and serial casting by physiotherapists [14]. This finding has necessitated the importance of enhancing awareness of series of remedial approaches to the disabling clinical conditions particularly during antenatal session.

Majority of participants easily identified alcohol, some un-prescribed medications and smoking during pregnancy as risk factors to BD. For instance, 324(73.1%) participants in this study were aware that smoking during pregnancy would possibly lead to BD. This finding is in agreement with the study by Hackshaw et al., where significantly positive association was found between maternal smoking and BD [15]. It follows therefore that irrespective of the environment, majority of pregnant women recognize smoking to be detrimental to health. The previous study did not indicate advanced maternal age as a risk factor to the occurrence of BD. In the present study, 192(43.3%) indicated advanced age of the mother as a possible risk factor for BD incidence. Again, the present findings were in agreement with the outcome of the study by Tan et al. which revealed that 80.7% and 71.7% of postnatal and antenatal mothers respectively were aware that the risk of Down syndrome increases with maternal age of community-dwellers in Singapore [16].

One hundred and eighty-seven participants (42.2%) correctly identified that consumption of salt fortified with iodine will reduce the risk of BD. This falls short of expectation given the documentation by the March of Dimes Foundations that iodine deficiency, alcohol and anti-epileptic drugs are known to increase the risk of BD in pregnant women [17,18]. The sampled participants may not have been abreast with iodine as an essential ingredient in reducing the risk of BD but may be aware about the general importance of iodated salt which might have informed their responses. Large proportion of participants, 371 (83.7%) agreed that regular medical checkup with health care personnel throughout the pregnancy period will most likely reduce the risk of giving birth to a child with BD based on their knowledge on preventive measures. This response conforms to the key recommendation by National Birth Defects Prevention Network on the importance of regular consultation with health care providers [3].

Indeed, various perceptions and beliefs abound with regard to child birth and pregnancy in most African countries. It was found that 48.1% of the sampled participants attributed BD to supernatural factors. Thus, eventhough the participant’s knowledge about BD was generally moderate, the existing underlying beliefs and perceptions could be the overriding factors that influenced the level of knowledge of the sampled participants irrespective of their socio-demographic profile. There is the need therefore to profile some of the existing public beliefs and opinions about BD with a view to juxtapose them into awareness campaign programme for pregnant women especially during antenatal visit. The National Birth Defects Prevention Network advocated a serries of strategies for pregnant women in a bid to increase their awareness about BD [3]. These measures include, management of chronic marternal illness, consultation with healthcare providers before taking any medication, consulting health care providers regularly and avoidance of alcohol, smoking and the use of illicit drugs.

The outcomes of this study is limited by the questionnaire employed to determine the participants’ metric outcome of their knowledge on BD. Slight variation could exist in the interpretations of some items either correctly or otherwise depending on the individuals’ conjecture and this might affect the external validity of the results.

## Conclusion

Within the limitation of this study however, it is concluded that the sampled participants generally possess moderate knowledge on specific and overall knowledge while they had high knowledge on the risk factors about BD. Also, there seem to be a disconnect between the acquired knowledge about BD and their socio-demographic profile. The perceived beliefs about the occurrence of BD appear to have a significant influence on their overall knowledge base. This study thus suggests a need for more awareness campaign about pregnancy and BD among Ghanaian pregnant women during antenatal visits and other relevant public health campaigns by correcting traditional beliefs and perception regarding the defects.

## Appendix

### Birth defects questionnaire for pregnant women

Knowledge of Birth Defects among Ghanaian Pregnant Women attending Antenatal Clinics at Three Selected Hospitals

Section A: socio-demographics

Kindly provide the following informations:

1. Age (years)

2. Education level:

a. Primary

b. Secondary

c. Tertiary

d. None

3. Occupation

4. How many pregnancy have you had so far?

a. One

b. Two

c. Three

d. Four

e. Five

f. Six

g. Seven

5. How many antenatal clinic visits have you made in total?

a. Less than 10

b. 10 to 19 times

c. 20 to 29 times

d. 30 or more.

Section B

Knowledge on birth defects and its risk factors

For the purpose of this questionnaire, birth defects, congenital malformation/disorder means the same thing.

Participants’ specific knowledge on birth defects

Which of the following correctly describes birth defects

1. It is a disease acquired by pregnant women

a. Yes

b. No

c. I don’t know

2. It can be acquired by baby developing in the womb

a. Yes

b. No

c. I don’t know

3. It can be transmitted by contact with affected individuals

a. Yes

b. No

c. I don’t know

4. Most birth defects are preventable

a. Yes

b. No

c. I don’t know

5. Most birth defects can be treated or managed medically

a. Yes

b. No

c. I don’t know

6. Deformities from congenital malformation can be reduced through physiotherapy

a. Yes

b. No

c. I Don’t know

Participants’ knowledge on risk factors and prevention

Kindly respond to the following questions as accurate as possible:

1. Will alcohol consumption during pregnancy increase your risk of giving birth to a child with BD?

a. Yes

b. No

c. I don’t know

2. Will the use of some unprescribed medications increase your risk of giving birth to a child with BD?

a. Yes

b. No

c. I don’t know

3. Will smoking before and during pregnancy increase your risk of giving birth to a child with BD?

a. Yes

b. No

c. I don’t know]

4. Will advance maternal age (≥40 years) increase the risk of giving birth to a child with BD?

a. Yes

b. No

c. I don’t know

5. Will consumption of iodated salt during pregnancy reduce your chance of giving birth to a child with BD?

a. Yes

b. No

c. I don’t know

6. Will regular checkups throughout the pregnancy period reduce your chances of giving birth to a child with BD?

a. Yes

b. No

c. I don’t know

7. In your own opinion, what do you think is responsible for Birth Defects? (You may choose multiple answers)

a. Supernatural factors

b. Giving birth to many children

c. Living an immoral life style

d. Medical reason

e. Eating some forbidden foods during pregnancy

f. Other please specify

## Competing interest

The authors declare that they have no competing interests.

## Authors’ contributions

AIB: Responsible for the design of the manuscript for intellectual presentation. He also contributed to the collection, management, analysis and interpretation of data. AAA: Assisted in the collection, management, analysis and interpretation of data. JNAQ: Contributed to the management and interpretation of data. He also assisted in editing the manuscript for its final presentation. AH: Participated in the collection of data and final editing of the manuscript. All authors read and approved the final manuscript.

## Pre-publication history

The pre-publication history for this paper can be accessed here:

http://www.biomedcentral.com/1471-2393/13/45/prepub
